# *Plasmodium vivax* infection: atypical memory B cells are expanded and associated with the persistence of Duffy binding protein II (DBPII) antibody response

**DOI:** 10.1186/1475-2875-13-S1-P52

**Published:** 2014-09-22

**Authors:** Flora S Kano, Barbara AS Lima, Michaelis L Tang, Pedro AC Costa, Cor JF Fontes, Bruno M Sanchez, Roberto S Rocha, Irene S Soares, Cristiana FA Brito, Lis Antonelli, Luzia H Carvalho

**Affiliations:** 1 Centro de Pesquisas René Rachou/FIOCRUZ, Belo Horizonte, MG, Brazil; 2 Universidade Federal do Mato Grosso, Cuiabá, MT, Brazil; 3 Instituto de Pesquisas Leônidas e Maria Deane/FIOCRUZ, Manaus, AM, Brazil; 4 Universidade de São Paulo, São Paulo, SP, Brazil

## 

Antibody responses generated during malaria infection represent an important component of acquired clinical immunity. Despite that, B cell subpopulations induced by the *Plasmodium vivax* (Pv) infection remains largely unknown. Here, we demonstrated that activated as well as “atypical” memory B cells (MBCs) are expanded in peripheral blood of *Pv*-exposed individuals, but their frequencies were not associated with acute infection. Aiming to investigate the association between peripheral B cells subsets and *Pv*-specific antibodies, we further followed-up 34 individuals exposed to *P. vivax* in the Brazilian Amazon area, an area of markedly unstable malaria transmission; after three cross-sectional survey (at 6-months intervals), ELISA-detected specific IgG (AMA-1, MSP1-19, DBPII) allowed the classification of those individuals as non-responder (NR), temporary (TR) or persistent responder (PR). For AMA-1 and MSP1-19 serological groups, the frequencies of MBCs (classical and atypical) and plasma cells (PCs) were similar among the groups. For DBPII group, we found a trend toward decreases classical MBCs according to the antibody response (NR>TR>PR). On the other hand, the frequencies of atypical MBCs increased according to the presence and persistence of DBPII antibody response (PR>TR>NR). Altogether, these results showed that atypical MBCs are expanded in Pv-exposed individuals (infected and non-infected), and it seems to be associated with the persistence of DBPII antibody response. Although preliminary, these results suggest that atypical MBCs contribute in generation of malarial antibody responses and provide insight into the role of atypical MBCs in *P. vivax* malaria immunity.

